# Optimization of heat inactivation protocols for *Orientia* and *Rickettsia* species

**DOI:** 10.1038/s41598-025-06728-w

**Published:** 2025-07-01

**Authors:** Artharee Rungrojn, Mohammad Yazid Abdad, Sandhya Dhawan, Jantana Wongsantichon, Stuart D. Blacksell

**Affiliations:** 1 Mahidol Oxford Tropical Medicine Research Programme, Faculty of Tropical Medicine, Mahidol University, 420/6 Rajvithi Road, Bangkok, 10400 Thailand; 2 Centre for Tropical Medicine and Global Health, Nuffield Department of Medicine, University of Oxford, Oxford, OX3 7LG UK

**Keywords:** *Orientia*, *Rickettsia*, Heat inactivation, Bacteria, Biological techniques, Microbiology

## Abstract

Heat treatment, or thermal disinfection, is one of the simplest and most widely used methods for microbial inactivation. Proper heat inactivation protocols are essential to ensure the safe transportation and handling of infectious materials, particularly for organisms in risk group 3, such as *Rickettsia* and *Orientia*. In this study, we examined the inactivation of four bacterial species—*Orientia tsutsugamushi*, *Rickettsia typhi*, *Rickettsia conorii*, and *Rickettsia honei*—at temperatures of 56 °C, 80 °C, and 90 °C for durations of 5, 15, 30, and 60 min. Observations were made at 0, 1, 3, 7, 10, and 14 days post-infection (dpi) to assess bacterial infectivity by monitoring bacterial DNA copies in newly infected cells. Our results indicate that 56 °C for 5 min was the minimum temperature and time required to inactivate *O. tsutsugamushi*, *R. typhi*, *R. conorii*, and *R. honei*. *O. tsutsugamushi* exhibited a higher reduction factor at 56 °C compared to *R. typhi*, *R. conorii*, and *R. honei*. Additionally, a strong inverse correlation between incubation time and log10 reduction factor was observed for *O. tsutsugamushi* and *R. typhi*, underscoring the importance of both time and temperature in effective heat treatment. However, no such correlation was observed for *R. conorii* and *R. honei*. These findings highlight the variable responses of bacteria to heat, emphasizing the need for pathogen-specific approaches in inactivation protocols. Optimizing heat treatment strategies based on these insights is critical for enhancing biosafety and ensuring effective pathogen eradication.

## Introduction

Rickettsial diseases in humans are primarily caused by members of the genera *Rickettsia* and *Orientia*, which belong to the family Rickettsiaceae. These gram-negative, obligate intracellular bacteria have dimensions of 0.3–0.5 µm in diameter and 0.8–2.0 µm in length^1^. *Rickettsia* and *Orientia* are significant public health concerns, particularly in the Asia–Pacific region^2^. Cultivation of these pathogens requires antibiotic-free cell culture systems^3^ or media supplemented with Penicillin G and Streptomycin^4^. Additionally, their propagation for research purposes must be conducted in laboratories with Heightened Control Measures, including Biosafety Level 3 facilities^5–7^.

Biosafety and biocontainment measures for *Orientia* spp. and other similar rickettsial organisms have traditionally been adapted from practices established for *Rickettsia* spp. While *Orientia* and *Rickettsia* spp. are believed to exhibit comparable susceptibilities to physical and chemical inactivation, such assumptions may be flawed, as they fail to account for critical differences between the two genera. The practice of extrapolating biosafety protocols from one genus to another risks ignoring essential risk-based principles. Current biosafety practices for *Rickettsia* and *Orientia* spp. often rely on a "one size fits all" approach, which lacks robust evidence to justify bypassing tailored risk assessments^6^.

Access to high-containment laboratories has posed a significant challenge for many research groups studying *Orientia* and *Rickettsia* spp. Consequently, attempts to share materials between laboratories or handle these infectious organisms necessitate inactivation to ensure safe handling^8^. Typically, a temperature of 56 °C is used for the heat inactivation of biological materials and cultures^9,10^; however, at our institution, the Mahidol Oxford Tropical Medicine Research Unit (MORU), a higher temperature range of 80–90 °C has been routinely employed for inactivation based on empirical observations^11^.

Comparative studies on the inactivation of rickettsiae, particularly *Orientia* and *Rickettsia* spp., remain limited. This study examines the efficacy of heat inactivation by directly applying heat to the cell culture grown and purified bacteria via a water bath at temperatures commonly referenced in the literature. Various temperatures and exposure times were tested on *Orientia tsutsugamushi*, *Rickettsia typhi*, *Rickettsia conorii*, and *Rickettsia honei* as representative species of the two genera. The success of inactivation or loss of bacterial infectivity was determined by the absence of bacterial growth in in vitro culture following heat treatment. Successfully inactivated bacteria will allow for the safe handling and transport of infectious substances outside biosafety containment, facilitating their use in various laboratory procedures and sharing of reagents.

## Material and methods

### Cell line and multiplicity of infection (MOI) of *Orientia tsutsugamushi* and *Rickettsia *spp.

The L929 cell line (European Collection of Authorised Cell Cultures (ECACC) 85,011,425) was maintained at 80–90% confluence in RPMI 1640 medium (Gibco™, Thermo) supplemented with 10% fetal bovine serum (FBS, Sigma®) without antibiotics. Monolayers were cultured in T25 cell culture flasks at 37 °C in a humidified atmosphere containing 5% CO_2_. The multiplicity of infection (MOI) was determined using aliquots of *O. tsutsugamushi*, *R. typhi*, *R. conorii*, and *R. honei* obtained from frozen stocks stored in sucrose phosphate glutamine (SPG) buffer (0.218 M sucrose, 3.76 mM potassium dihydrogen phosphate, 7.1 mM dipotassium hydrogen phosphate, 4.9 mM monosodium L-glutamic acid) at − 80 °C. A dilution series consisting of four tenfold dilutions (1:10 to 1:10,000) was prepared from the stock to achieve an estimated MOI of 10. DNA copies were evaluated by qPCR. The recovery percentage was evaluated in triplicate. Recovery of *O. tsutsugamushi* was assessed by calculating the ratio of undiluted bacteria recovered post-thaw to the amount initially frozen.

$${\text{Recovery}}_{{{\text{OT}}}} \left( \% \right) = \frac{{{\text{Amount of}}\;O.\;tsutsugamushi\;{\text{copies per mL}}_{{{\text{undiluted}}}} }}{{{\text{Amount of}}\;O.\;tsutsugamushi\;{\text{copies per mL}}_{{{\text{frozen}}}} }} \times 100$$

L929 monolayers were infected with *O. tsutsugamushi*, *R. typhi*, *R. conorii*, and *R. honei*. After five days of infection, the culture medium was discarded, and the cell layer was washed once with 1X phosphate-buffered saline (PBS, Gibco Thermo Fisher Scientific, UK). The infected cells were harvested by scraping with a sterile inoculating loop into 2 mL of 1X PBS and disrupted using a bullet blender (BBX24B, Bullet Blender Blue, Next Advance, USA) to release intracellular bacteria. Cellular debris was removed by centrifugation at 300 × g for 5 min, and the released intracellular bacteria in the supernatant were pelleted by centrifugation at 20,000 × g for 5 min. The purified bacterial pellet was resuspended in fresh 1X PBS and used for subsequent inactivation.

### Heat inactivation preliminary and experiment

#### Orientia tsutsugamushi

**Fig. 1:**
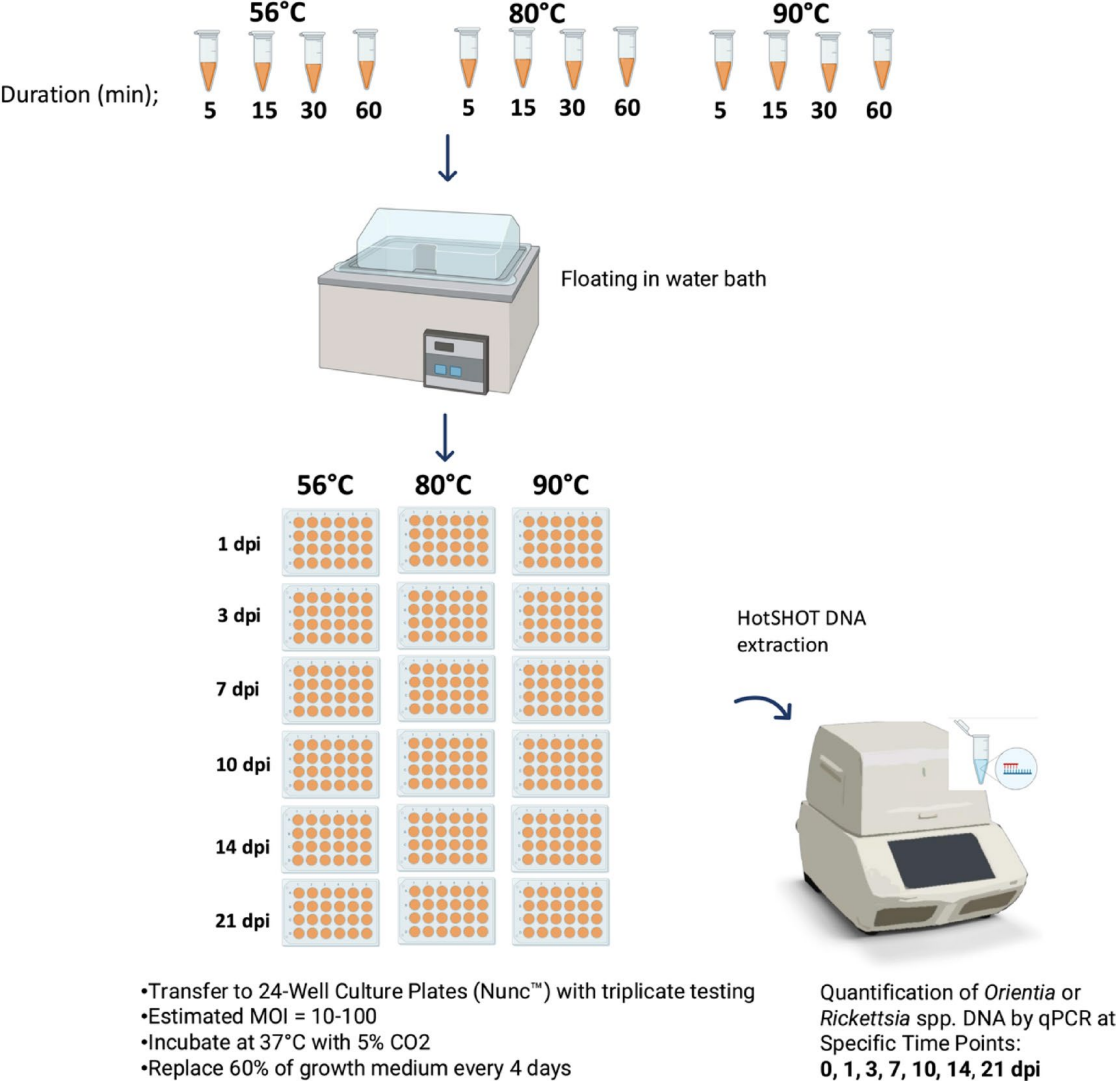
Preliminary experiments and workflow for the inactivation of *O. tsutsugamushi* and *R. typhi*. Created in BioRender. Rungrojn, A. (2025) https://BioRender.com/s50w157.

The purified organisms (*Orientia* and *Rickettsia*) were used in this study as previously described^12^. In brief, preliminary heat inactivation tests were conducted on *O. tsutsugamushi* harvested from cell cultures. The scraped bacterial-infected L929 suspension was transferred to a 1.5 mL safe-lock tube (Eppendorf Safe-Lock®, UK) and processed using a bullet blender (Next Advance, USA) for 1 min. After centrifugation at 300 × g for 3 min to remove cell debris, the supernatant was transferred to a new tube and centrifuged again at 20,000 × g for 3 min to harvest bacterial cells. The final supernatant was discarded, and the bacterial pellet was resuspended in PBS. A 100 µL aliquot was taken for HotSHOT DNA extraction to confirm the presence of *O. tsutsugamushi* nucleic acid material (detailed in the ‘Quantification DNA Extraction’ section). Since the target genes used for qPCR-based nucleic acid detection in this study are single-copy per genome, DNA copy numbers were used to indicate bacterial quantity for subsequent MOI calculations. The remaining aliquots were heat-treated in a water bath (Memmert®, Germany) at three temperatures (56 °C, 80 °C, and 90 °C) for durations of 5, 15, 30, and 60 min. Heat-inactivated samples were added to 24-well culture plates (Nunc™) containing 80% confluent L929 cell monolayers at an MOI of 10–100^9^. The plates were incubated in a 5% CO_2_ environment at 35 °C for 1, 3, 7, 10, 14, and 21 days. To replenish the medium, approximately 60% of the growth medium was replaced every 4 days. At each specified day post-infection (dpi), the L929 monolayer in selected wells was washed twice, scraped, and disrupted using a bullet blender homogenizer. The presence of propagated intracellular viable bacteria was monitored by detecting *O. tsutsugamushi* DNA via qPCR. Positive controls were untreated *O. tsutsugamushi* samples, while negative controls consisted of uninfected L929 cell cultures. Based on the findings, 56 °C for 5, 15, 30, and 60 min were selected for triplicate testing. Observations were conducted at 0, 1, 3, 7, 10, and 14 dpi to monitor *O. tsutsugamushi* DNA (see Fig. 1).

#### *Rickettsia typhi*, *Rickettsia conorii,* and *Rickettsia honei*

During the preliminary testing phase, *R. typhi* was used as the representative organism for *Rickettsia* spp., with protocols identical to those employed for *O. tsutsugamushi.* After completing preliminary tests, triplicate heat inactivation experiments were conducted for *R. typhi, R. conorii,* and *R. honei* at 56 °C and 80 °C for 5, 15, 30, and 60 min. Observations were recorded at 0, 1, 3, 7, 10, and 14 dpi to monitor the heat inactivation process and evaluate its effectiveness (see Fig. 1).

### DNA extraction and quantitative PCR analysis

DNA extraction was performed using the HotSHOT method (Hot Sodium Hydroxide and Tris)^13^. Briefly, 100 µL of each sample was centrifuged at 20,000 × g for 5 min. The resulting bacterial pellet was resuspended in 20 µL of alkaline lysis reagent (25 mM sodium hydroxide, 0.2 mM EDTA, pH 12.0) and incubated at 95 °C for 30 min. Samples were then cooled to room temperature, and an equal volume of neutralizing reagent (40 mM Tris–HCl, pH 5.0) was added.

The copy numbers of *O. tsutsugamushi* and *Rickettsia* spp. in each seed stock were determined using Quantitative PCR, which has been shown to be a comparable quantification method to the plaque assay, as described previously^14^. Real-time PCR with a TaqMan assay was used to quantify bacterial DNA. DNA extracted by the HotSHOT method from *O. tsutsugamushi*, *R. typhi*, *R. conorii*, and *R. honei* was amplified using primers and probes targeting the 47 kDa periplasmic serine protease (htrA) gene for *O. tsutsugamushi* and the 17 kDa surface antigen gene for *Rickettsia* spp. (see Table 1)^15,16^. Standard curves were generated from serial dilutions (10^1^ to 10^7^ copies/µL) of plasmid DNA suspensions for *O. tsutsugamushi* and *R. typhi*. Quantitative PCR was conducted with Platinum Quantitative PCR SuperMix-UDG (Invitrogen Life Technologies, Paisley, UK) using 0.2 µM primers and 0.1 µM probe for *O. tsutsugamushi* and 0.4 µM primers and probe for *Rickettsia* spp. Reactions were performed in a Bio-Rad CFX Manager system (version 3.1) under the following conditions: 50 °C for 2 min, 95 °C for 2 min, followed by 45 cycles of 95 °C for 15 s and 60 °C for 30 s.

**Table 1:** Oligonucleotide sequences of primers and probes used in this study.

Primer and probe	Organism	Oligonucleotide 5′ → 3’	Product Size (bp)	References
OtsuFP630	*O. tsutsugamushi*	AACTGATTTTATTCAAACTAATGCTGCT	118	^16^
OtsuRP747		TATGCCTGAGTAAGATACRTGAATRGAATT		
OtsuPR665		6-FAM-TGGGTAGCTTTGGTGGACCGATGTTTAATCT-BHQ1		
R17K128F2	*Rickettsia* spp.	GGGCGGTATGAAYAAACAAG	111	^15^
R17K238R		CCTACACCTACTCCVACAAG		
R17K202TaqP		6-FAM-CCGAATTGAGAACCAAGTAATGC-BHQ1		

### Reduction factor (RF) calculation

RF values were calculated using the equation R = log10(NiNf)R = log10(NfNi), where NiNi represents the initial microbial population (> 1,000) and NfNf represents the final population (< 1).

### Statistical analysis and software

These analyses used bacterial copy number per mL as the key response variable. Data were tested for normal distribution before statistical processing. Arithmetic means and standard deviations (SD) were calculated to summarize the data. Statistical analyses were carried out using IBM SPSS Statistics (version 29) and R (v4.3.1 “Beagle Scouts”), and primary data visualizations were prepared using GraphPad Prism 10.

## Results

### MOI and freeze–thaw effects on the survival of *O. tsutsugamushi*

**Fig. 2:**
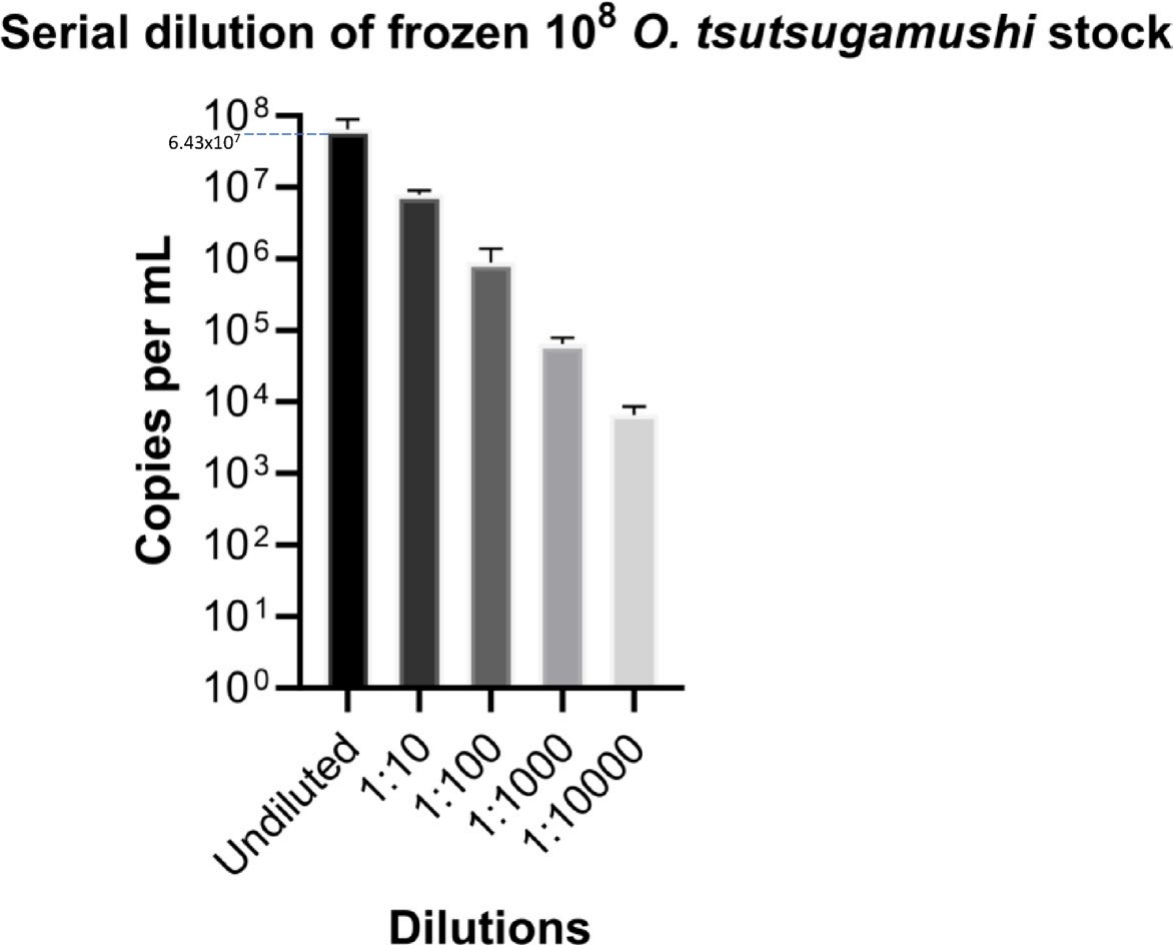
Post-thaw viability of cryopreserved *O. tsutsugamushi* bacteria. “Undiluted” refers to the initial concentration of cells frozen. Ten-fold serial dilutions were prepared at 1:10, 1:100, 1:1,000, and 1:10,000.

This study employed Cryopreservd *O. tsutsugamushi, R. conorii*, and *R. honei* as primary seed stocks for bacterial propagation. The effect of the freeze–thaw cycle was evaluated using 10^8^ seed stock of *O. tsutsugamushi*. After thawing the frozen stock, the remaining concentration of the undiluted stock was 6.43 × 10^7^; as shown in Fig. 2, 64.3% of *O. tsutsugamushi* was successfully recovered from the original tube. With this freeze–thaw effect, the remaining concentration was used to calculate an MOI of 10 to inoculate onto confluent L929 monolayers in T25 flasks to prepare bacterial cells prior to heat treatment. To ensure consistency, all samples were incubated on L929 cells for 5 days post-infection, maintaining identical conditions for *O. tsutsugamushi*, *R. typhi*, *R. conorii*, and *R. honei*.

### Heat inactivation

#### *O. tsutsugamushi* heat inactivation preliminary and experiment testing

**Fig. 3:**
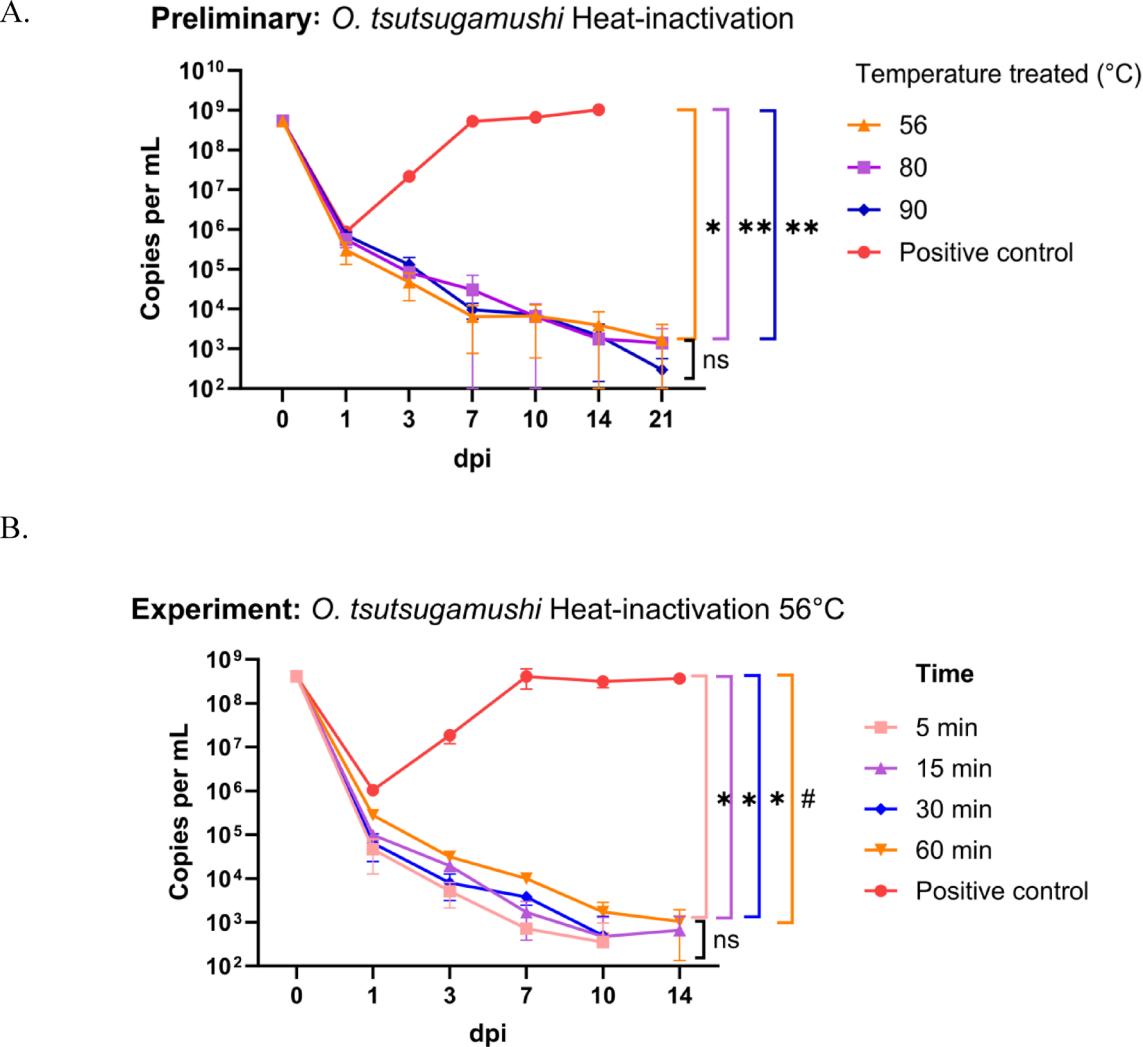
Assessment of bacterial growth for *O. tsutsugamushi* following heat inactivation. (**A**) Preliminary growth curves after heat treatment at 56 °C, 80 °C, and 90 °C for durations of 5, 15, 30, and 60 min. (**B**) Growth curves following heat inactivation at 56 °C across various incubation times. Statistical analysis was conducted using the Kruskal–Wallis test for comparisons between the positive control and heat-treated groups, followed by Dunn’s post-hoc test for pairwise comparisons of heat-treated groups. Statistical significance is indicated as: *P* < 0.001 (*), *P* = 0.002 (**), *P* = 0.001 (#), and *ns* (not significant).

Heat inactivation of *O. tsutsugamushi* Karp was assessed at 56 °C, 80 °C, and 90 °C for durations of 5, 15, 30, and 60 min. Observations were recorded at 0, 1, 3, 7, 10, 14, and 21 days post-infection (dpi). At all three temperatures, the infectivity of *O. tsutsugamushi* began to decline after 1 dpi. Preliminary results identified 56 °C as the minimum temperature required to inactivate *O. tsutsugamushi* Karp-infected L929 cells (Fig. 3A). The optimal incubation time was subsequently evaluated at 56 °C based on these findings. *O. tsutsugamushi* Karp was heat-treated for 5, 15, 30, and 60 min, then re-cultured and observed at 1, 3, 7, 10, and 14 dpi. The MOI for reinoculation was 10–100, corresponding to a DNA input of 2.085 × 10^6^ total copies per well. DNA levels decreased at 1 dpi across all heat-treated samples, including the positive control. At all time points (5, 15, 30, and 60 min), *O. tsutsugamushi* DNA exhibited consistent log reductions over 14 dpi, with levels dropping below 100 copies per mL by the end of the observation period. In contrast, the positive control demonstrated a statistically significant increase in DNA levels after 1 dpi, saturating at 7 dpi (Fig. 3B). The negative control consistently showed no detectable DNA, and these results were excluded from the graph.

#### Rickettsia spp. heat inactivation

**Fig. 4:**
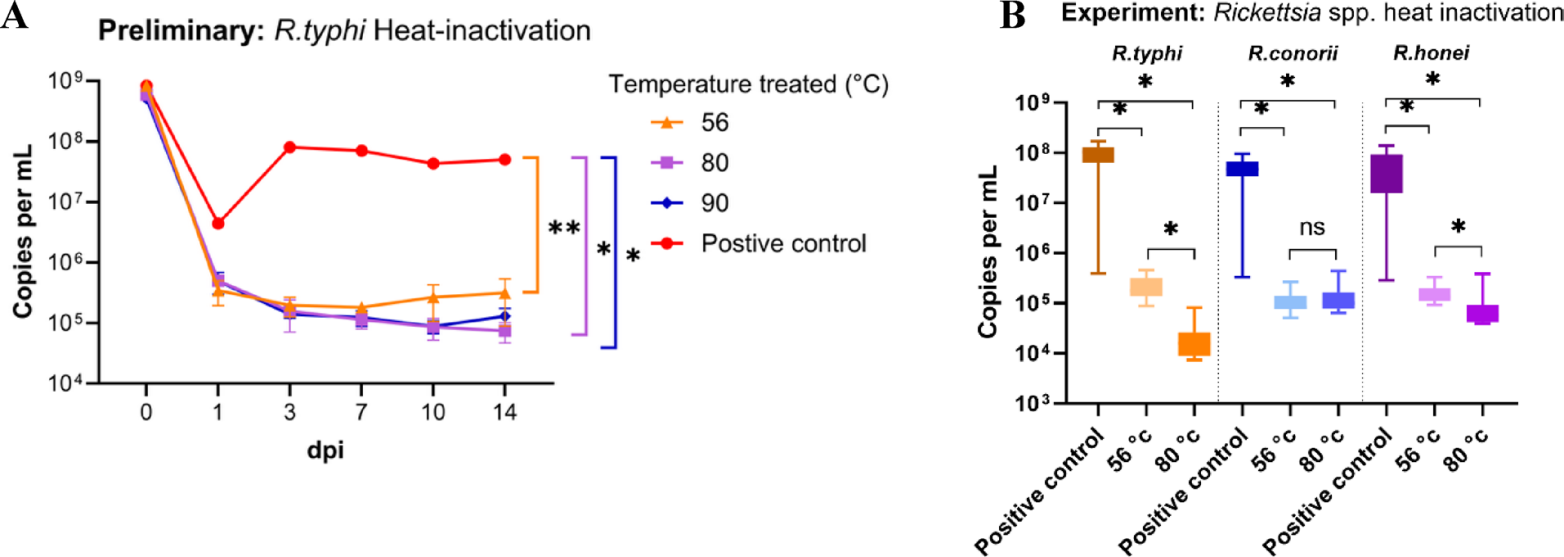
Bacterial growth assessment of *R. typhi* following heat inactivation. (**A**) Preliminary growth studies of *R. typhi* after exposure to 56 °C, 80 °C, and 90 °C. (**B**) Experimental studies performed in triplicate for *R. typhi*, *R. conorii*, and *R. honei* treated at 56 °C and 80 °C. Statistical significance was determined using the Kruskal–Wallis test, followed by Dunn’s post-hoc test. Results are denoted as follows: *P* < 0.01 (*), *P* = 0.022 (**), and *ns* for non-significant outcomes.

Heat inactivation of *R. typhi* was conducted as a model during the preliminary testing phase for *Rickettsia* spp. Our findings showed that *R. typhi* infectivity began to decline gradually after 1 dpi at temperatures of 56 °C, 80 °C, and 90 °C, with a continuous decrease observed through 14 dpi. We established that 56 °C was the minimum temperature required to inactivate *R. typhi*-infected L929 cells. Statistical analysis using the Kruskal–Wallis test followed by Dunn’s post hoc test revealed significant differences between the positive control and heat-treated groups at 56 °C, 80 °C, and 90 °C (*P* = 0.022, *P* < 0.01, *P* < 0.01, respectively). However, no significant difference was observed between 80 and 90 °C treatments (Fig. 4A).

Based on these preliminary results, we focused on temperatures of 56 °C and 80 °C to further investigate the efficacy of heat inactivation. Repeated experiments were conducted in triplicate using *R. typhi*-, *R. conorii*-, and *R. honei*-infected L929 cells, with observations recorded through 14 dpi. When comparing heat-treated groups at 56 °C and 80 °C with the positive control, a statistically significant mean reduction of approximately 3 logs in rickettsial DNA was observed (*P* < 0.001). Additionally, significant differences were detected between the 56 °C and 80 °C treatments for *R. typhi* and *R. honei* (*P* < 0.001) (Fig. 4B).

### Temperature effects on DNA Copy numbers and RF analysis

#### DNA copy numbers

**Fig. 5:**
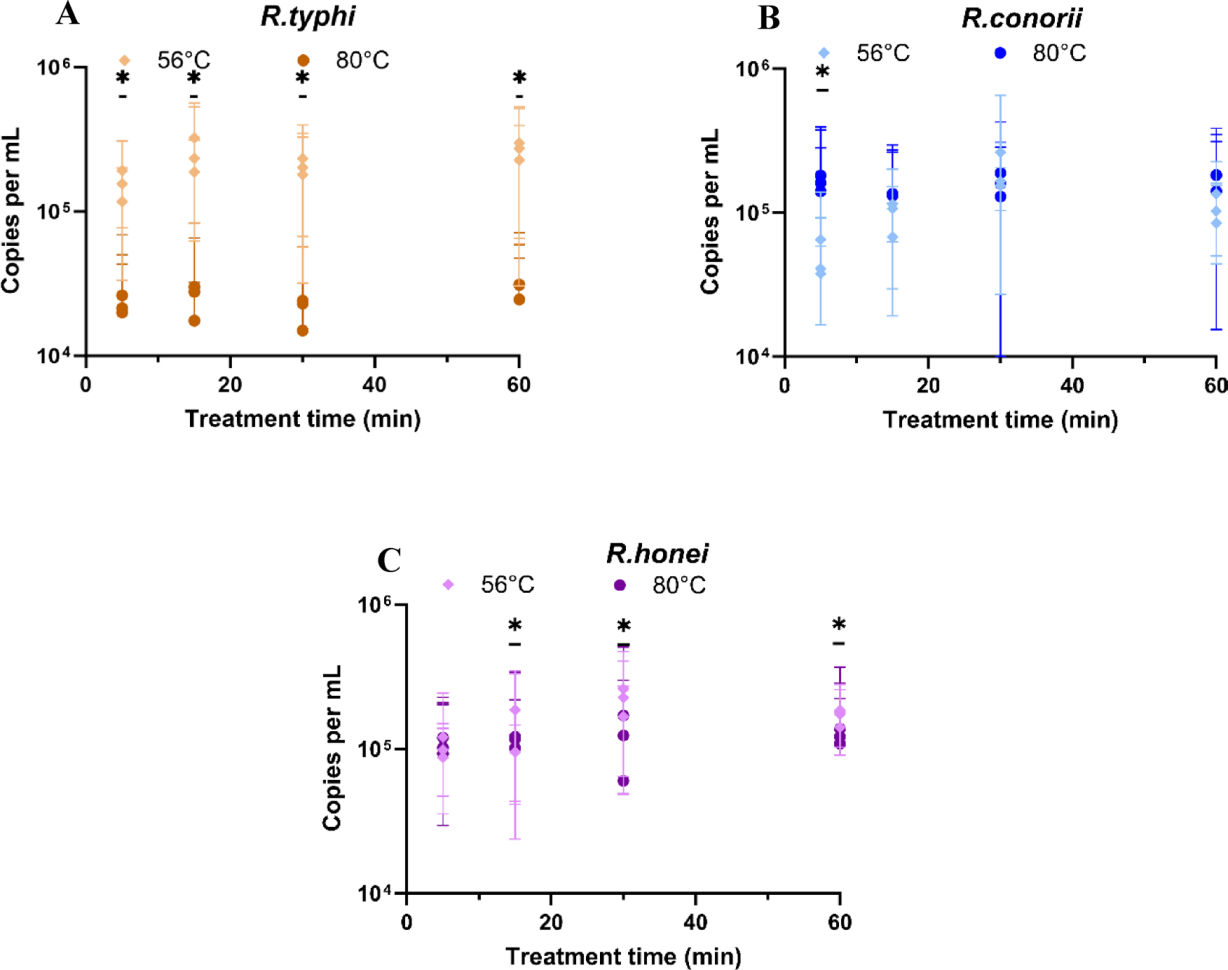
Heat treatment effects on *Rickettsia* species (*R. typhi*, *R. conorii*, and *R. honei*), comparing DNA copy numbers at 56 °C and 80 °C across incubation times (5, 15, 30, and 60 min). Graphs include 95% confidence intervals. Statistical significance, as determined by the Mann–Whitney U test, is indicated by an asterisk (*).

The data for *R. typhi* revealed statistically significant differences (*P* < 0.001) in DNA copy numbers between the two temperature treatments (56 °C and 80 °C) across all time points. A greater reduction in *R. typhi* bacterial copies was observed at 80 °C compared to 56 °C (Fig. 5A). For *R. honei*, DNA copy numbers decreased significantly at 80 °C compared to 56 °C, with significant differences at the 15, 30, and 60-min time points (*P* = 0.015, 0.001, 0.007, respectively) (Fig. 5B). These findings suggest higher temperatures are more effective at reducing *R. honei* bacterial load. The data for *R. conorii* also showed significant differences in DNA copy numbers at 80 °C versus 56 °C at the 5-min time point (*P* < 0.001). However, confidence intervals overlapped at later time points, indicating variable responses to temperature treatments over time (Fig. 5C).

#### Reduction factor analysis at 56 °C

**Fig. 6:**
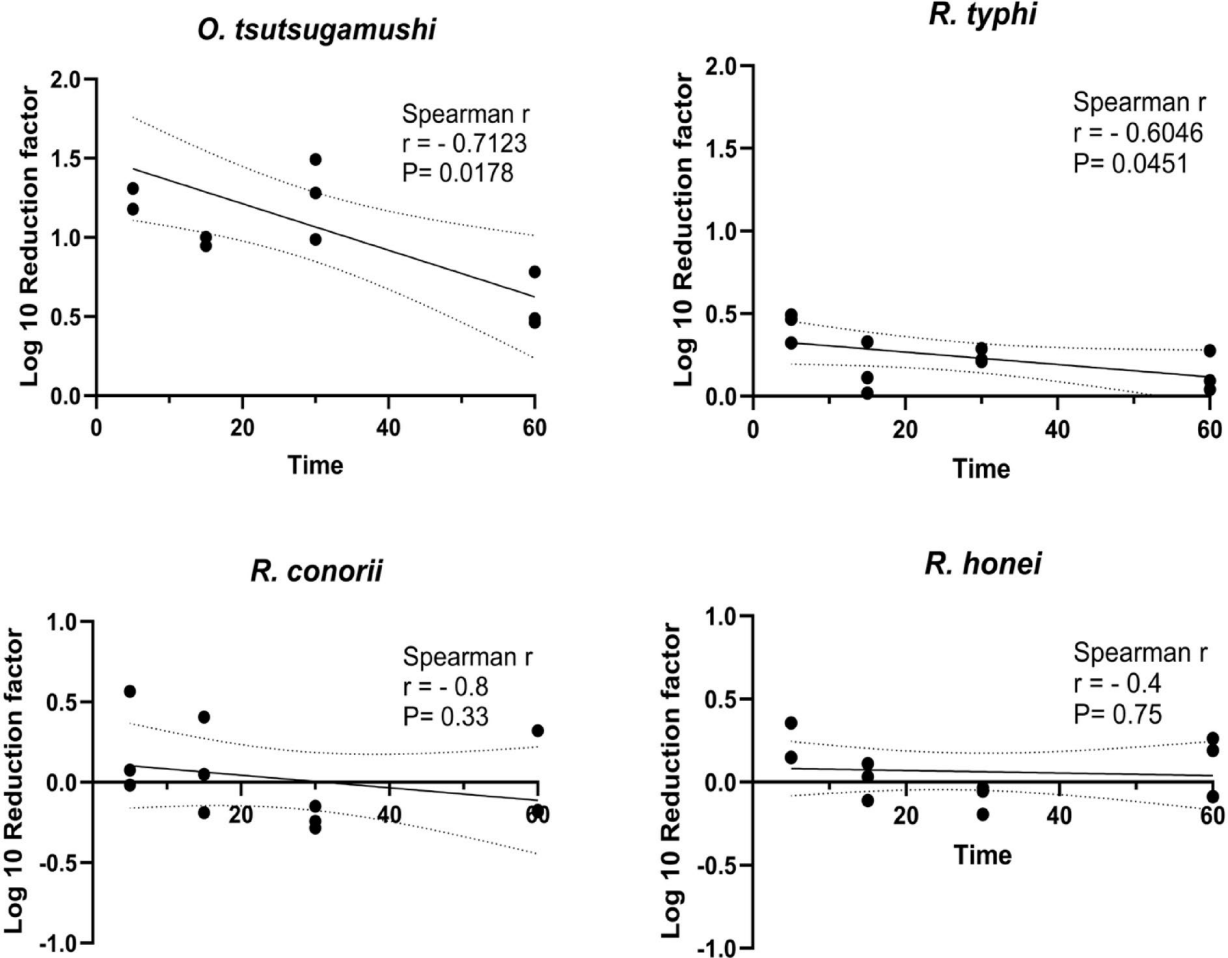
Correlation between heat treatment duration and log reduction of bacterial DNA copy numbers. The analysis demonstrates a negative linear relationship between the bacterial DNA copy numbers for the 56 °C heat treatment group and the positive control group at 1 dpi (days post-inoculation) across different incubation times. The reduction factor, calculated using the R package, was based on bacterial reinoculation results at 1 dpi. All experiments were conducted with a sample size of *n* = 3. The relationship between treatment duration and the log_10_ reduction factor was assessed using the Spearman correlation test. The dotted line indicates the 95% confidence interval.

The RF was used to measure the effectiveness of the heat treatment at 56 °C for inactivating *O. tsutsugamushi*, *R. typhi*, *R. conorii*, and *R. honei*. Analysis at 1 dpi revealed a significant negative correlation between increased incubation time and log_10_ RF for *O. tsutsugamushi* (R^2^ = 71.23, *P* = 0.0178) and *R. typhi* (R^2^ = 60.46, *P* = 0.0451). However, no significant correlation was observed for *R. conorii* (*P* = 0.33) or *R. honei* (*P* = 0.75) (Fig. 6). These results highlight differences in susceptibility to heat treatment among these organisms.

## Discussion

Effective microbial inactivation is critical for ensuring biosafety in laboratory settings, particularly for risk group 3 pathogens such as *O. tsutsugamushi* and *Rickettsia* spp. While heat inactivation is widely used, existing protocols are adapted mainly from research on *Rickettsia* despite key biological differences between these genera. This study provides an in-depth assessment of heat inactivation across multiple *Rickettsia* species and *O. tsutsugamushi*, demonstrating that a minimum temperature of 56 °C for 5 min is sufficient to inactivate these organisms. These findings reinforce the importance of pathogen-specific inactivation protocols, as assumptions based on one genus may not always apply to another.

Several inactivation methods have been evaluated for rickettsiae, including treatments with formalin, Qiagen AVL buffer, β-propiolactone, and heat^17^. Formalin and β-propiolactone make the pathogen unsuitable for downstream applications due to serious adverse effects, while AVL buffer contains detergents that compromise bacterial structural integrity^17^. Our findings indicate that treatment at 56 °C, 80 °C, and 90 °C provides comparable inactivation effects, with 56 °C for 5 min being the minimum temperature and duration required to inactivate *O. tsutsugamushi*, *R. typhi*, *R. conorii*, and *R. honei*. Previous studies have employed temperatures such as 56 °C for 30 min^9,10^, 90 °C for 30 min^11^, and 100 °C for 30 min^18^ for inactivation. In an animal model, one study also utilized riboflavin and light treatment depending on the pathogen’s resistance to inactivate *O. tsutsugamushi* in blood components^19^. Our findings establish that 56 °C for 5 min is the minimum condition required for effective inactivation of *O. tsutsugamushi*. The study indicates that heat-treated bacteria have lost their infectivity, as evidenced by reduced bacterial DNA copy numbers in newly infected cells. However, it does not confirm complete inactivation or loss of viability.

Preliminary inactivation tests for *R. typhi* revealed no differences in results between 80 and 90 °C. Consequently, 56 °C and 80 °C were selected for the rickettsiae inactivation experiments. A significant reduction in DNA quantity was observed for *R. typhi* and *R. honei* with prolonged incubation at 80 °C. Previous studies suggest that higher temperatures can alter the tertiary structure and antigenicity of rickettsial pathogens^20^, consistent with findings by Frickmann and Dobler (2013), which reported that *R. honei* begins to inactivate at 56 °C after 5 min^17^.

For *R. conorii*, no significant differences were observed between inactivation at 56 °C and 80 °C. Among the four incubation durations tested, a 5-min period was statistically significant for measurable bacterial viability reduction, but extending the incubation time did not further affect pathogen viability. Although the copy numbers of *R. conorii* at 14 dpi were slightly lower following heat treatment at 56 °C compared to 80 °C, the difference was statistically insignificant (*P* = 0.3, data not shown). This suggests that bacterial reduction is comparable at both temperatures.

The RF is a critical metric for evaluating bacterial reduction effectiveness^21^. *O. tsutsugamushi* displayed a higher log_10_ RF compared to *R. typhi*, *R. conorii*, and *R. honei*, indicating greater bacterial load reduction at 56 °C for *O. tsutsugamushi*. This enhanced efficacy may result from the absence of adaptive mechanisms, such as thermostable lipopolysaccharides and group-specific antigens, which are present in the genus *Rickettsia* ^22–24^. Furthermore, the negative correlation between extended incubation time at a constant temperature and log_10_ RF likely reflects the survival of heat-resistant bacterial subpopulations or the induction of stress responses during prolonged incubation. These mechanisms may enhance bacterial resilience to subsequent heat exposure, reducing the overall effectiveness of thermal treatment^25,26^. A significant negative correlation was identified between incubation time and log_10_ RF for *O. tsutsugamushi* and *R. typhi*, indicating that extended incubation times reduce RF values for these organisms. In contrast, no statistically significant relationship was observed for *R. conorii* and *R. honei*. These results suggest that the effectiveness of heat inactivation at 56 °C is both pathogen-specific and influenced by incubation time, emphasizing the importance of tailoring heat treatment processes to the characteristics of individual bacteria.

In this study, SPG freezing buffer was used for *O. tsutsugamushi* cryopreservation cultures, building on prior research demonstrating its effectiveness in preserving culture viability before freezing at − 80 °C^12^. In line with previous studies, we used an MOI of 10–100 for *O. tsutsugamushi* and *Rickettsia* species^27,28^. Our observations indicated that freeze–thaw cycles had a significant impact on bacterial population dynamics^29,30^. Specifically, after a single freeze–thaw cycle, the DNA of *O. tsutsugamushi* decreased by 35.7%, corresponding to a reduction of 0.19 log. These findings underscore the importance of considering freeze–thaw effects on microbial populations^31^.

This study is subject to certain limitations. Inactivation testing focused solely on the Karp strain of *O. tsutsugamushi*. While the findings may be relevant to other strains, such as Kato, Gilliam, and UT76, this was not directly assessed. Similarly, the study included only three species of *Rickettsia*—*R. typhi*, *R. conorii*, and *R. honei*—with other species in the genus not evaluated. While members of the same genus are often biologically similar, further research is advised to confirm effective inactivation, especially for species exhibiting significant genomic or biological diversity. Another limitation of this study is the exclusive use of the L929 murine fibroblast cell line. Given that infectivity and replication dynamics of *Rickettsia* spp. may vary depending on the host cell line, particularly between mammalian and insect-derived cells. Further comparative studies using tick or mosquito cell lines may provide additional insight into pathogen-specific responses to heat inactivation.

This study provides empirical evidence supporting the use of 56 °C for just 5 min as the minimum effective condition for *O. tsutsugamushi* and *Rickettsia* spp. inactivation. The observed differences in heat susceptibility between these genera reinforce the need for pathogen-specific inactivation protocols rather than relying on assumptions from related bacteria. These findings have direct biosafety implications, particularly for laboratories handling rickettsial pathogens, and contribute to ongoing discussions on refining international biosafety guidelines for risk group 3 organisms. Future research should focus on the long-term stability of heat-inactivated bacteria for downstream applications, such as serological assays and antigen-based detection methods. Additionally, it is important to investigate how heat inactivation affects protein structure and immunogenicity, as these factors could have significant implications for vaccine development and the preparation of diagnostic reagents.

## Data Availability

All data generated or analysed during this study are included in this published article.
